# The safety and efficacy of PDE5-inhibitors-vardenafil on treating diabetes mellitus erectile dysfunction: A protocol for systematic review and meta analysis

**DOI:** 10.1097/MD.0000000000018361

**Published:** 2019-12-20

**Authors:** Jia He, Xiao Li, Heng-Heng Dai, Ji-Sheng Wang, Hai-Song Li, Xiao-Jun Zhang, Ping Wang, Dong Zhang, Ling-Yan Zuo, Ning Xie, Ying Li

**Affiliations:** a Department of Pharmacy; b Department of Science And Education, Beijing Longfu Hospital, Being; c Scientific Research Office; d Department of General Medicine, Peking Union Medical College Hospital, Chinese Academy of Medical Sciences; e Department of Medical Devices Management, Beijing Dongcheng District Community Health Service Management center; f Department of General Surgery, Beijing Longfu Hospital; g Department of Andrology, Dongzhimen Hospital, Dongcheng District, Hai Yun Cang on the 5th ZIP, Beijing, China; h Department of Andrology, The First Affiliated Hospital of Henan University of Chinese Medicine, Zhengzhou, China.

**Keywords:** diabetic mellitus erectile dysfunction, PDE5 inhibitors, systematic review, vardenafil

## Abstract

**Background:**

Diabetic mellitus erectile dysfunction (DMED) refers to erectile dysfunction (ED) secondary to diabetes. As people's lifestyle changes and the population ages, the incidence of DMED continues to increase. Many clinical trials have proven that PDE5-inhibitors-vardenafil has a significant effect in the treatment of Diabetic mellitus erectile dysfunction. In this systematic review, we aim to evaluate the effectiveness and safety of PDE5-inhibitors-vardenafil for Diabetic mellitus erectile dysfunction.

**Methods:**

We will search PubMed, Cochrane Library, AMED, EMbase, WorldSciNet; Nature, Science online and China Journal Full-text Database (CNKI), China Biomedical Literature CD-ROM Database (CBM), and related randomized controlled trials included in the China Resources Database. The time is limited from the construction of the library to February 2019.We will use the criteria provided by Cochrane 5.1.0 for quality assessment and risk assessment of the included studies, and use the Revman 5.3 and Stata13.0 software for meta-analysis of the effectiveness, recurrence rate, and symptom scores of Diabetic mellitus erectile dysfunction.

**Ethics and dissemination:**

This systematic review will evaluate the efficacy and safety of PDE5-inhibitors-vardenafil for treating Diabetic mellitus erectile dysfunction. Because all of the data used in this systematic review and meta-analysis has been published, this review does not require ethical approval. Furthermore, all data will be analyzed anonymously during the review process Trial.

**Trial registration number:**

PROSPERO CRD42018095185.

## 1 Introduction

Diabetic mellitus erectile dysfunction (DMED) refers to erectile dysfunction (ED) secondary to diabetes. It is characterized by persistent or repetitive penile erection and insufficient hardness or sufficient time to be satisfied. The phenomenon of completing sexual activity.^[1,2]^ It is a type of diabetic sexual dysfunction.^[3]^ With the improvement of people's living standards, the incidence of diabetes, especially type 2 diabetes, has been increasingly increased, and the complications it brought cover multiple organs of the human body.^[4,5]^ Erectile dysfunction is one of its common complications. Surveys in the United Kingdom, the United States, and other countries have shown that the incidence of erectile dysfunction in the normal population is 0.1% to 18%. However, the incidence of erectile dysfunction in diabetic patients has increased nearly three-fold compared with the normal population, and tends to be younger.^[6]^ Studies have shown that the number of people with ED in diabetes has reached 71% in the past 10 years. Diabetes ED patients often have severe symptoms and are a type of refractory ED, which seriously affects the quality of lives of the diabetic patients.^[7]^

Pharmacotherapy is the primary treatment for ED, including PDE5 inhibitors, androgen therapy, and vasoactive agents.^[8–11]^ Phosphodiesterase-5 (PDE5) inhibitors, the first-line oral drugs recommended by World Health Organization (WHO) for the treatment of ED, have also begun to be widely used in the treatment of DMED, included Sildenafil, Tadalafil, Vardenafil, and so on.^[12–14]^ The medicine can mainly inhibit PDE5, expressed in the corpus cavernous, to increase cGMP concentration in vascular smooth muscle cells, decrease intracellular calcium concentration, cause smooth muscle relaxation and increase cavernous blood flow which could improve erectile situation.^[15]^ Among them, vardenafil is especially widely used in the treatment of DMED. Research reports that the application of vardenafil in recent years has been increasing year by year. Studies have shown that PDE5-inhibitors-vardenafil treatment of DMED can improve the International Index of Erectile Function-5 (IIEF-5) and sexual success rate in a considerable number of patients.^[16,17]^ Although meta-analyses have shown that PDE5-inhibitors-vardenafil can safely and effectively treat ED, whether they are still safe and effective for DMED with more complex etiologies remains to be assessed.^[18,19]^ Therefore, this review hopes evaluate the efficacy and safety of PDE5-inhibitors-vardenafil in the treatment of DMED to provide the newest evidence for clinical.

## 2 Methods

This is a systematic review and ethical approval was not necessary.

### 2.1 Study registration

This systematic review protocol has been registered on PROSPERO as CRD42018095185. (https://www.crd.york.ac.uk/prospero/display_record.php?ID=CRD42018095185)

### 2.2 Eligibility criteria

#### 2.2.1 Type of study

Take PDE5-inhibitors-vardenafil or combined with other effective interventions as main treatment, including randomized controlled trials of the control group (effective methods other than PDE5-inhibitors-vardenafil). Language is limited in Chinese and English. Non-randomized controlled trials, quasi-randomized controlled trials, case series, case reports, and crossover studies will be excluded.

#### 2.2.2 Participants

Men with a history of diabetes who match the Diagnostic Criteria for Diabetes: Refer to the American Diabetes Association (ADA) Diabetes Care Guidelines. The diagnosis is ED after diabetes, and the International Index of Erectile Function 5 (IIEF-5) score is <21. The course of ED is ≥ 3 months. The patient must be at least 18 years of age. The sexual partners of the patients are fixed. The group is well balanced when enrolled.

#### 2.2.3 Types of interventions

##### 2.2.3.1 Experimental interventions

The treatment group will use the PDE5-inhibitors-vardenafil, with no limited of the dose and frequency of the medicine. The trial period requires more than 1 course of treatment.

##### 2.2.3.2 Control interventions

As for the control interventions, who accepted simple western medicine can be used as a control intervention or did not get any treatment as a blank control would be adopted. However, once they had accepted the therapy of PDE5-inhibitors-vardenafil, the trials will be rejected.

#### 2.2.4 Outcomes

The primary outcome measurement will be assessed using the International Erectile Function Index.

(1) Healing: IIEF-5 score ≥22 points after treatment;
(2) Significant effect: IIEF-5 score <22 points after treatment, score improvement≥60%;
(3) Effective: IIEF-5 score <22 points after treatment, points improved <60%, but≥30%;
(4) invalid: IIEF-5 score <22 points after treatment, score improvement <30%.

The secondary outcome measurement will be assessed according to the PDE5-inhibitors-vardenafil syndrome scoring criteria.

(1) Healing: The clinical symptoms and signs of PDE5-inhibitors-vardenafil disappear or disappear, and the syndrome score is reduced by ≥90%;
(2) Markedly effective: the clinical symptoms and signs of PDE5-inhibitors-vardenafil are obviously improved, the syndrome score is reduced by ≥60%;
(3) Effective: PDE5-inhibitors-vardenafil clinical symptoms Signs and signs have improved, syndrome points reduced by <60%, but ≥30%;
(4) Invalid: Chinese clinical symptoms and signs have not improved, or even worse, syndrome scores reduced by <30%.Integral variation formula (Nimodipine method:[(pre-treatment score − post-treatment score) ÷ pre-treatment score]×100%.

#### 2.2.5 Data source

##### 2.2.5.1 Electronic searches

The electronic search database includes PubMed, Cochrane Library, AMED, EMbase, WorldSciNet, Nature, Science online and China Journal Full-text Database (CNKI), China Biomedical Studies CD-ROM Database (CBM), and China Resources Database. The clinical research studies on the treatment of Diabetic mellitus erectile dysfunction with Chinese medicine published in domestic and foreign biomedical journals from the establishment of the library to October 2019 was searched. Based on the standards of the Cochrane Collaboration Workbook of the International Evidence-Based Medicine Center, a manual and computer-based method is used to conduct related studies. The search terms include: PDE5-inhibitors-vardenafil, Diabetic mellitus erectile dysfunction, impotence. According to the characteristics of different databases, comprehensive search using keyword and keyword. All search results are determined after multiple searches. We will follow the references included in the studies in order to incorporate relevant studies as much as possible to avoid missed detection.The search term in the Chinese database is the translation of the above word. The complete PubMed search strategy is summarized in Table 1.

**Table 1:**
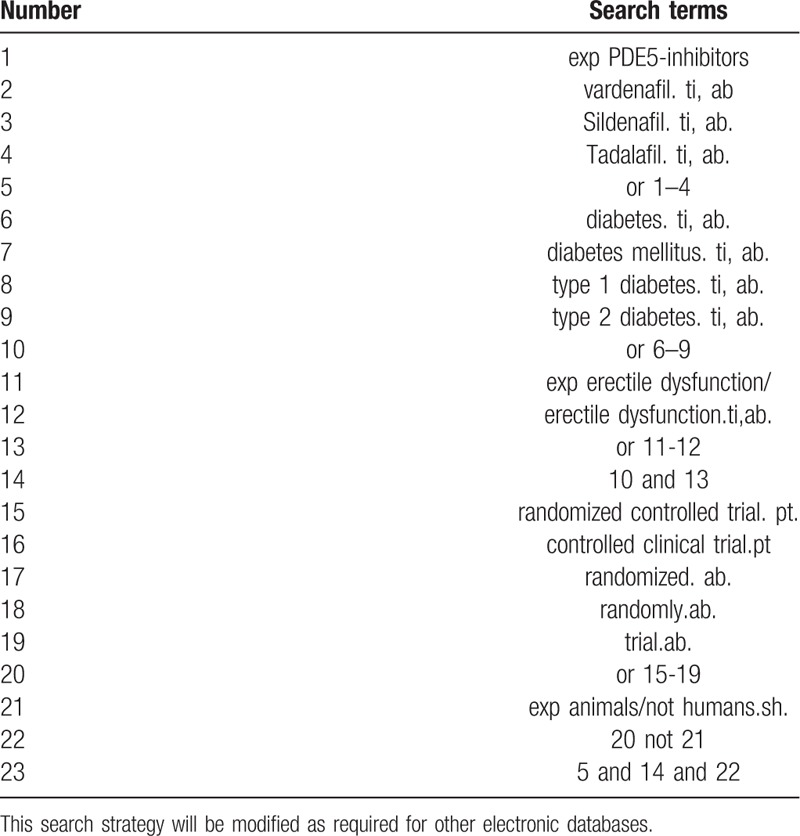
Search strategy used in PubMed database.

#### 2.2.6 Data collection and analysis

**Figure 1:**
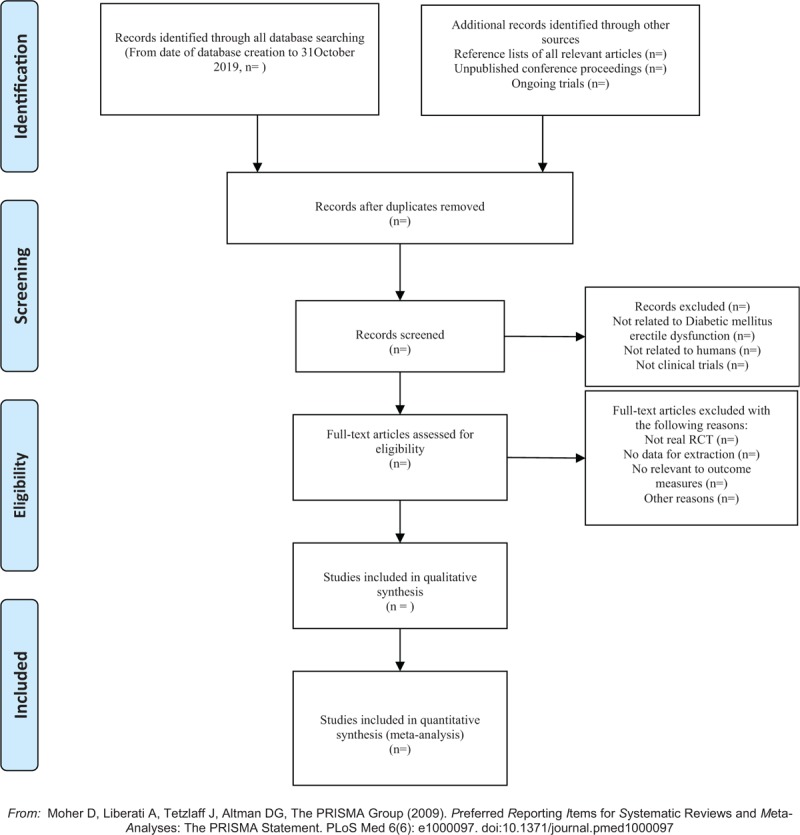
The PRISMA flow chart.

(1) Applying the EndnoteX7 software to manage the included references. Two qualified evaluators independently screened the titles and abstracts of the selected studies, excluding duplicates and documents that did not significantly conform to the study.
(2) The second screening of the studies: Screening out unqualified studies: such as case report, theoretical discussion, and non-conformance of interventions. After preliminary evaluation, carefully read the remaining studies to further screen out the unqualified studies: such as ordinary control study, no control group, no random grouping, no outcome index, and data mine equivalent.
(3) For the studies that cannot be determined whether it can be included in the study, it is decided by 2 researchers. If the opinions are not uniform, please ask a third-party expert. A clinical randomized controlled trial (RCT) is finally included in the study.
(4) Information and data extraction for the final included documents. The extracted data and information will include the test methods of the study, the basic information of the included cases, the observation period, the intervention methods of the treatment group and the control group, the observation indicators, and the test results. (Fig. 1)

#### 2.2.7 Risk of bias

The studies quality assessment applies the bias risk assessment tool recommended by Cochrane to evaluate the quality of all included studies and the risk of bias. The assessment will include sequence generation, allocation concealment, blinding of participants, personnel and outcome assessors, incomplete outcome data, selective outcome reporting, and other sources of bias. The risk of high and low bias is expressed as “high risk” and “low risk”, respectively. The information provided in the study is inaccurate or does not provide sufficient information for the bias assessment to be expressed as “unclear risk”. The above content evaluation is independently evaluated by 2 researchers. If there are different opinions, the discussion will be conducted. If there are still differences, consult the third appraiser. Otherwise, consult with the Cochrane Professional Group.

#### 2.2.8 Statistical analysis

The meta-analysis in this study will use Rev Man5.3 and Stata13.0 statistical software. Heterogeneity tests will be used for the included experimental studies. The numerical variable will be expressed as the normalized mean difference (SMD) with a confidence interval (CI) of 95%. The heterogeneity of each pairwise comparison will be tested by Chi-square test (test level α = 0.1). If there is no heterogeneity, a fixed effect model will be used. If there is significant heterogeneity between a set of studies, we will use a random effects model (REM) for meta-analysis. We will explore the reasons for the existence of heterogeneity from various aspects such as the characteristics of the subjects and the degree of variation of the interventions. The source of heterogeneity is further determined by means of sensitivity analysis.

#### 2.2.9 Publication bias

If a result of a meta-analysis contains more than 10 articles and above, we will use a funnel plot to test the risk of publication bias. Quantitative methods such as Begg testing and Egger testing will be used to help assess publication bias in the application.

##### 2.2.9.1 Quality of evidence

The GRADE method will be used to assess the quality of evidence for key outcomes. This assessment will be conducted through a Guideline Development Tool. (GRADEpro GDT, https://gradepro.org/)

## 3 Discussion

In recent years, with the changes in people's lifestyles and the aging of the population, the incidence of diabetes has increased. Diabetes has a variety of chronic complications, including heart disease, high blood pressure, stroke, and ED. Among them, diabetes is most closely related to ED and is the leading cause of organic ED.^[20,21]^ About half of diabetic patients have ED. With the deepening of understanding of diabetes and its complications, the trials and clinical reports of PDE5-inhibitors-vardenafil treatment of DMED have gradually increased. Phosphodiesterase-5 (PDE5) inhibitors, the first-line oral drugs recommended by World Health Organization (WHO) for the treatment of ED, have also begun to be widely used in the treatment of DMED, included Sildenafil, Tadalafil, Vardenafil, and so on.^[22–24]^ The medicine can mainly inhibit PDE5, expressed in the corpus cavernous, to increase cGMP concentration in vascular smooth muscle cells, decrease intracellular calcium concentration, cause smooth muscle relaxation and increase cavernous blood flow which could improve erectile situation. Among them, vardenafil is especially widely used in the treatment of DMED. Research reports that the application of vardenafil in recent years has been increasing year by year.^[25,26]^

Therefore, we will compare the effectiveness and safety of PDE5-inhibitors-vardenafil in the treatment of DMED by applying systematic evaluation and meta-analysis. The results of this study can provide a possible ranking for PDE5-inhibitors-vardenafil treatment of DMED. We hope that the results will provide clinicians with the best options for treating DMED and provide them with research directions. Although we will conduct a comprehensive search in this study, languages other than Chinese and English will be restricted, which will lead to some bias. In addition, the relevant studies on PDE5-inhibitors-vardenafil treatment of DMED are small and the overall quality is low, which may affect the authenticity of this study. Therefore, we hope that in the future, we will have a more rigorous and reasonable multi-center randomized controlled trial to explore the clinical efficacy of PDE5-inhibitors-vardenafil in the treatment of DMED, so that the conclusion is more objective and reasonable.

## Author contributions

**Data curation:** Jia H, Xiao-Jun Zhang

**Formal analysis:** Jia H, Ping Wang, Dong Zhang

**Funding acquisition:** Ping Wang, Ling-Yan Zuo

**Project administration:** Jia H, Xiao Li

**Supervision:** Ning Xie, Ying Li

**Validation:** PW, Ling-Yan Zuo

**Writing – original draft:** Jia H, Ping Wang, Ning Xie

**Writing – review & editing:** Ying Li, Xiao-Jun Zhang, Heng-Heng Dai
